# Generation of Synthetic Non-Homogeneous Fog by Discretized Radiative Transfer Equation

**DOI:** 10.3390/jimaging11060196

**Published:** 2025-06-13

**Authors:** Marcell Beregi-Kovacs, Balazs Harangi, Andras Hajdu, Gyorgy Gat

**Affiliations:** 1 Faculty of Informatics, University of Debrecen, 4028 Debrecen, Hungary; harangi.balazs@inf.unideb.hu (B.H.); hajdu.andras@inf.unideb.hu (A.H.); 2 Institute of Mathematics, University of Debrecen, 4028 Debrecen, Hungary; gat.gyorgy@science.unideb.hu

**Keywords:** radiative transfer equation, fog synthesis, discretization, physical modeling, image augmentation, inhomogeneous media

## Abstract

The synthesis of realistic fog in images is critical for applications such as autonomous navigation, augmented reality, and visual effects. Traditional methods based on Koschmieder’s law or GAN-based image translation typically assume homogeneous fog distributions and rely on oversimplified scattering models, limiting their physical realism. In this paper, we propose a physics-driven approach to fog synthesis by discretizing the Radiative Transfer Equation (RTE). Our method models spatially inhomogeneous fog and anisotropic multi-scattering, enabling the generation of structurally consistent and perceptually plausible fog effects. To evaluate performance, we construct a dataset of real-world foggy, cloudy, and sunny images and compare our results against both Koschmieder-based and GAN-based baselines. Experimental results show that our method achieves a lower Fréchet Inception Distance (−10% vs. Koschmieder, −42% vs. CycleGAN) and a higher Pearson correlation (+4% and +21%, respectively), highlighting its superiority in both feature space and structural fidelity. These findings highlight the potential of RTE-based fog synthesis for physically consistent image augmentation under challenging visibility conditions. However, the method’s practical deployment may be constrained by high memory requirements due to tensor-based computations, which must be addressed for large-scale or real-time applications.

## 1. Introduction

Realistic simulation of atmospheric optical effects, such as fog, haze, and mist, remains a significant challenge in computer graphics and computer vision. These phenomena emerge from complex interactions between light and suspended particles, leading to scattering, absorption, and multiple scattering behaviors. Accurate modeling of such effects is essential for various applications, including virtual reality, autonomous navigation, and visual effects production.

Among these, fog simulation plays a pivotal role in enhancing depth perception and environmental realism. Conventional methods, including empirical models like Koschmieder’s Law and data-driven approaches such as Generative Adversarial Networks (GANs), often assume homogeneous fog distributions and rely on simplified light scattering models. While efficient, these approaches fail to capture the spatial and angular variability observed in natural foggy environments.

In this work, we propose a physics-based algorithm for the generation of synthetic fog that is based on the Radiative Transfer Equation (RTE). The RTE provides a comprehensive framework for modeling light propagation in participating media, incorporating spatial heterogeneity, anisotropic scattering, and multi-path radiative interactions. By discretizing the RTE, we develop a numerically tractable formulation that enables the synthesis of spatially varying fog while preserving computational efficiency.

Our method is evaluated using a custom-built dataset comprising paired cloudy and foggy images, allowing for quantitative benchmarking against both classical models and GAN-based baselines. The results demonstrate that our approach produces more structurally consistent and perceptually realistic fog effects than existing techniques, highlighting its potential for use in safety-critical and high-fidelity rendering scenarios.

The rest of this paper is organized as follows. Section 2 reviews prior work in fog simulation and image-space radiative transfer. Section 3 introduces the theoretical background, including the Radiative Transfer Equation (RTE) and its discretized numerical formulation. In Section 4, we describe the construction and annotation of our real-world fog dataset. Section 5 details the proposed fog synthesis pipeline and the optimization of physical parameters. Section 6 presents the experimental evaluation, followed by discussion in Section 7 and concluding remarks in Section 8.

## 2. Related Works

Research on fog simulation methods has evolved along two primary lines: physically grounded models based on radiative transfer theory and data-driven synthesis using generative models. Although the former ensures physical fidelity, it is often limited by computational demands and domain-specific assumptions. The latter, while efficient and visually convincing, tends to overlook the underlying optical physics that governs fog formation and perception. In this section, we review representative works from both approaches and position our method accordingly.

The Radiative Transfer Equation (RTE) serves as the theoretical backbone for modeling light propagation in turbid media such as fog. Studies such as [1,2] employed deterministic and GPU-based methods, respectively, to approximate multi-scattering and extinction in large-scale fog environments. Duthon et al. [3] and classic references like Fowler and Sung [4] utilize Mie theory to define the scattering phase functions under varying particle distributions.

Although physically accurate, these methods are typically designed for scientific computation or remote sensing, and thus they are impractical for image-space fog synthesis due to their high computational cost.

Several studies have investigated the inversion of the RTE to retrieve fog properties from image data. Krayem et al. [5] addressed fog particle size distribution through RTE inversion, while Guo et al. [6] extended this to coupled thermal radiation problems. These approaches are conceptually relevant but not targeted at photorealistic synthesis.

Simplified atmospheric models based on Koschmieder’s law remain popular for real-time applications, such as in automotive vision systems. Tarel and Hautière [7] developed an enhancement method tailored to heterogeneous fog in road scenes, while earlier works [8] introduced single-image contrast restoration via atmospheric veil estimation. These models benefit from speed and simplicity but assume uniform fog and planar geometries.

Generative models offer a flexible alternative for fog augmentation. Sun et al. [9] proposed a single-image depth-from-defocus approach combined with scattering models. Recent advances such as StarGAN-v2 [10] enable multi-weather simulation (fog, night, rain) using domain translation. However, these models lack physical transparency and offer limited parameter control, making them suboptimal for structure-preserving fog generation.

Weather-specific generative models have also emerged. Abbas and Babahenini [11] proposed a GAN-based approach for fog synthesis in forest environments, which achieves photorealism but struggles with complex geometries and GPU resource demands. Similarly, Lin et al. [12] introduced a weather translation model using modular attention and segmentation, capable of fog simulation, yet lacking physical interpretability.

To address data scarcity in rare conditions, Gong et al. [13] developed Analogical Image Translation (AIT), which performs zero-shot domain transfer using relational analogies between known style pairs. This method enables fog simulation without requiring target-style examples but remains style-driven and lacks parameter control.

More recently, diffusion-based methods have shown promise in hazy image generation. Zhang et al. [14] introduced Glow-Diffusion, a two-stage diffusion pipeline that incorporates physical priors such as atmospheric point spread and glow flare, achieving state-of-the-art realism at the cost of interpretability and controllability.

On the physics-based side, Ramazzina et al. [15] proposed ScatterNeRF, a neural inverse rendering model that disentangles the scene and scattering medium using volumetric radiative modeling. While accurate, it requires multi-view input and focuses on fog removal rather than controllable forward synthesis.

Ben-Daoued et al. [16] introduced SWEET, a physically grounded 3D simulator based on Monte Carlo ray tracing, developed specifically for evaluating perceptive automotive sensors (e.g., camera, LiDAR, radar) under foggy conditions. Their framework supports multiwavelength simulation and complex scene geometry, enabling the realistic modeling of electromagnetic propagation through fog. SWEET has been shown to outperform traditional image-space models like Koschmieder’s law, particularly under dense or night-time fog, and provides an essential tool for validating perception systems in extreme weather.

For applications in autonomous systems, Zhu et al. [17] developed a physically based fog and rain image generator for camera function testing. Their SIGM model combines geometric microparticle modeling with Mie-theory-based transmittance estimation, offering efficient, sensor-level simulation under adverse weather.

Simeon and colleagues [18] introduced a fast single-scattering model for simulating light sources in fog, providing closed-form approximations for cone and isotropic illumination using analytical solutions to the air–light integral. Although efficient, these methods assume homogeneous media and neglect multiple scattering.

In the context of dataset creation, Xie et al. [19] presented SynFog, a physically realistic synthetic fog dataset for training defogging models, incorporating sensor simulation and image signal processing to enhance realism. Their work confirms that camera-level modeling is essential for real-world generalization.

Unsupervised defogging approaches have also been proposed. Li et al. [20] introduced ZRDNet, a zero-reference defogging model combining physical decomposition and perceptual fusion. Though promising for fog removal without supervision, it does not provide mechanisms for fog generation or parameter tuning.

Domain adaptation methods such as UCDA-Flow by Zhou et al. [21] use unsupervised curriculum learning to bridge optical flow performance from clear to foggy scenes, emphasizing the importance of fog realism for downstream visual tasks.

We propose a discretized RTE-based fog generation method that combines the interpretability of physical models with the efficiency of image-based synthesis. Our approach supports inhomogeneous fog density and anisotropic scattering, and it outperforms both classical and learning-based baselines in structural realism and quantitative metrics.

To summarize, our work brings new results into the field of generating fog in the following ways:

- A physics-based fog synthesis framework is introduced, derived from the discretized Radiative Transfer Equation (RTE), enabling the simulation of spatially non-uniform and anisotropic scattering effects in image space;
- A numerically optimized algorithm is developed based on tensor contractions and precomputed interaction matrices, reducing computational complexity from $O(n^{5})$ to O(n) per iteration;
- A gradient-based parameter calibration scheme is formulated, allowing extinction and scattering coefficients to be learned from paired fog–cloudy image data;
- A curated dataset of annotated real-world images is constructed, incorporating subjective fog density labels and enabling comprehensive benchmarking against classical and generative baselines;
- Quantitative and qualitative evaluations indicate improved realism and structural fidelity compared to both Koschmieder’s model and CycleGAN-based baselines, as measured by the Fréchet Inception Distance (FID) and Pearson correlation.

## 3. Radiative Transfer Equation in Image-Space Fog Simulation

The radiative transfer theory (RTT) is a well-established framework in physics used to model the interaction between light and small particles in a medium. A central equation in this theory is the Radiative Transfer Equation (RTE), which describes the propagation of electromagnetic radiation—typically light—through participating media such as air.

The RTE represents three fundamental physical processes: absorption, scattering, and emission. Absorption describes radiation attenuation as a result of energy uptake by particles within the medium. Scattering refers to the redirection of light caused by interactions with those particles. Furthermore, emission describes the process by which the medium itself contributes radiative energy, often as a function of temperature.

By incorporating all three processes, the RTE enables a comprehensive assessment of the radiative energy balance in a medium. This is critical for accurately modeling visibility, illumination conditions, and other environmental parameters. The general form of the RTE is multidimensional, depending on spatial position, propagation direction, and wavelength (i.e., spectral composition) of the radiation.

### 3.1. Mathematical Formulation of Radiative Transfer

A comprehensive derivation of the Radiative Transfer Equation (RTE) for homogeneous media, including its application in visibility estimation, can be found in the doctoral thesis by Lenor [22]. While our work builds upon this theoretical foundation, we extend the model to handle spatially varying (non-homogeneous) fog distributions, which more accurately reflect real-world atmospheric conditions.

To this end, we begin by formulating the continuous RTE for a general scattering medium, introducing angular parametrization and the anisotropic phase function used in our model.

In the following, we analyze light propagation from the viewpoint of an observer and introduce the corresponding mathematical models required for the accurate description of scattering and absorption phenomena. Let the observer be located at point $M_{obs}\in R^{3}$, observing an object at point $M_{obj}\in R^{3}$ in the direction $\omega =(\omega_{1},\omega_{2})$ and at a distance *d*. Within a spherical coordinate system, these points can be expressed as follows:

(1) $$\begin{array}{cc} M_{obj} & =M_{obs}-P_{d,\omega }=-dP_{1,\omega }=-dP_{\omega }\\ \; & =-\left(x,y,z\right)=-\left(d\cos\left(\omega_{1}\right)\sin\left(\omega_{2}\right),d\sin\left(\omega_{1}\right)\sin\left(\omega_{2}\right),d\cos\left(\omega_{2}\right)\right), \end{array}$$

where the distance of $M_{obj}$ and $M_{obs}$ is −d≥0 and the vector $P_{\omega }\in S^{2}\subset R^{3}$ describes the observation direction of the observer, parameterized by the spherical angles $0\leq \omega_{1}\leq \pi$ and $0\leq \omega_{2}\leq \frac{\pi }{2}$. In spherical coordinates, the unit direction vector is given by

(2) $$P_{\omega }=\left(\cos\left(\omega_{1}\right)\sin\left(\omega_{2}\right),\sin\left(\omega_{1}\right)\sin\left(\omega_{2}\right),\cos\left(\omega_{2}\right)\right).$$

To accurately model the scattering process, we employ the Henyey–Greenstein phase function, which is a key component of the Radiative Transfer Equation (RTE). This function characterizes the angular scattering properties of particles within the medium and is defined as

(3) $$\phi \left(\omega ,\sigma \right)=\frac{1}{4\pi }\frac{1-g^{2}}{{\left(1-2g\langle P_{\omega },P_{\sigma }\rangle +g^{2}\right)}^{\frac{3}{2}}}.$$

Here, *g* denotes the asymmetry parameter, which typically lies in the range g∈[0.8,0.9]. The inner product $\langle P_{\omega },P_{\sigma }\rangle$ between the observation direction $\left(P_{\omega }\right)$ and the scattering direction $\left(P_{\sigma }\right)$ is computed as

(4) $$\langle P_{\omega },P_{\sigma }\rangle =\sin\left(\omega_{2}\right)\sin\left(\sigma_{2}\right)\cos\left(\omega_{1}-\sigma_{1}\right)+\cos\left(\omega_{2}\right)\cos\left(\sigma_{2}\right).$$

This expression plays a crucial role in the RTE, as it determines how radiation propagating in a given direction is redistributed due to scattering into other directions.

The general form of the Radiative Transfer Equation (RTE) can be expressed at a given point $M_{obj}$ as follows:

(5) $$M_{obj}=M_{obs}-rP_{\sigma }=-rP_{\sigma }$$

(6) $$\frac{\partial L}{\partial r}=K\left(r,\sigma \right)L\left(r,\sigma \right)-K_{s}\left(r,\sigma \right)\int_{S^{2}}L\left(r,\omega \right)\phi \left(\omega ,\sigma \right)d\left(P_{\omega }\right),$$

where K(r,σ) is the absorption coefficient, representing the energy loss due to absorption. $K_{s}\left(r,\sigma \right)$ is the scattering coefficient, which describes the scattering process. Finally, L(r,σ) represents the radiation intensity at a given distance *r* and direction σ. Here, $S^{2}$ denotes the set of all possible directions of incoming light.

These relationships are essential for modeling the propagation of light through scattering media such as fog. Such applications include meteorological visibility estimation, atmospheric optics, and astronomical and planetary research, and they are particularly critical in the automotive industry, where accurate estimation of visibility and light conditions is vital for the functioning of driver assistance systems.

To make (6) more intuitive and consistent with a physically meaningful parametrization, we perform a change of variable. Equation (6) models radiative propagation from the object toward the observer, which corresponds to a negative distance. However, this convention is counterintuitive in numerical implementations and physically less convenient. Therefore, we redefine the radiance function by a simple substitution, $L^{†}\left(r,\sigma \right):=L\left(-r,\sigma \right)$, for r>0, meaning that we measure the distance *r* forward from the object towards the observer. Taking the derivative of the reparametrized radiance function

(7) $$\frac{\partial L^{†}}{\partial r}\left(r,\sigma \right)=-\frac{\partial L}{\partial r}\left(-r,\sigma \right),$$

and substituting into (6), we obtain the following transformed Radiative Transfer Equation:

(8) $$-\frac{\partial L}{\partial r}=K\left(r,\sigma \right)L\left(r,\sigma \right)-K_{s}\left(r,\sigma \right)\int_{S^{2}}L\left(r,\omega \right)\phi \left(\omega ,\sigma \right)d\left(P_{\omega }\right).$$

From this point on, we drop the dagger and use *L* to denote the reparametrized function for simplicity.

The function L(r,σ) is defined as

(9) $$L\left(r,\sigma \right)=\left\{\begin{array}{cc} 0, & \mathrm{if}\;r>d\\ \mathrm{to}\;\mathrm{be}\;\mathrm{constructed}\;\mathrm{recursively}, & \mathrm{if}\;0<r\leq d\\ \mathrm{as}\;\mathrm{on}\;\mathrm{the}\;\mathrm{input}\;\mathrm{image}\;\mathrm{at}\;M_{obj}, & \mathrm{if}\;r=0. \end{array}\right.$$

Here, *d* denotes the distance between the observer and the object.

The image that we construct in the direction $P_{\sigma }$ at distance *d* from the observer is denoted by L(d,σ). This corresponds to the radiance as captured in the presence of fog or smog.

Consequently, the Radiative Transfer Equation at any point takes the form

(10) $$\frac{\partial L}{\partial r}=-K\left(r,\sigma \right)L\left(r,\sigma \right)+K_{s}\left(r,\sigma \right)\int_{S^{2}}L\left(r,\omega \right)\phi \left(\omega ,\sigma \right)d\left(P_{\omega }\right).$$

When both *K* and $K_{s}$ are assumed to be constant, the medium is referred to as homogeneous air. This simplification enables faster numerical implementation of the model, which is particularly beneficial in real-time systems, such as automotive camera applications.

Given the complexity and computational cost of the full Radiative Transfer Equation (RTE), practical applications often rely on simplified models. One of the most well-known approaches is the Koschmieder model, originally proposed in 1924 [23]. This model provides a significant simplification of the RTE while effectively capturing visibility reduction in homogeneous foggy atmospheres.

Koschmieder’s model is based on two fundamental assumptions:

1. Constant in-scattered radiance: $L_{\mathrm{in}}\left(p\left(s\right),\omega \right)\approx \mathrm{const}.$, implying that the in-scattered radiation does not vary spatially.
2. Homogeneous atmosphere: K=const. and $K_{s}=\mathrm{const}.$, meaning that both the absorption and scattering coefficients are uniform throughout the medium.

Under these assumptions, the line-integrated form of the RTE simplifies to

(11) $$L\left(d\right)=L_{0}e^{-Kd}+L_{\mathrm{air}}\left(1-e^{-Kd}\right),$$

where L(d) is the observed radiance at distance *d*, $L_{0}$ is the original radiance at d=0, *K* is the extinction coefficient accounting for both absorption and scattering effects, and, finally, $L_{\mathrm{air}}$ is the air–light parameter that represents the atmospheric background radiance defined as

(12) $$L_{\mathrm{air}}:=\frac{K_{s}}{K}\int_{S^{2}}L_{\mathrm{in}}\left(p\left(0\right),\omega \right)\psi \left(\sigma ,\omega \right)dS\left(\omega \right).$$

Koschmieder’s model is significant because it provides a closed-form expression for radiance attenuation in homogeneous fog. Combining exponential decay with a constant atmospheric background allows for the efficient computation of radiative intensity. This makes the model highly suitable for a range of practical applications, such as visibility estimation algorithms, where the homogeneous atmosphere assumption provides a reasonable approximation. Additionally, due to its low computational complexity, the model is used for real-time systems, including those used in the automotive industry.

### 3.2. Iterative and Discretized Approach

Previously, in Section 3.1, we presented the general formulation of the Radiative Transfer Equation (RTE), including anisotropic scattering and spatially varying media properties. Toward the end of that section, we introduced a simplified version—namely the Koschmieder model—which assumes a homogeneous atmosphere and constant in-scattered radiance.

However, real-world atmospheric conditions are often considerably more complex. The medium is typically non-homogeneous, and the scattering effects can be directionally dependent (anisotropic). Building on this foundation, we now propose an iterative numerical scheme designed to approximate the full RTE under such realistic, spatially varying conditions.

To facilitate numerical implementation, we discretize the continuous radiance function L(r,σ) over a uniform spatial and angular grid. Let ${\tilde{L}}_{j}\left(k_{1},k_{2}\right)$ denote the resulting radiance tensor, where *j* indexes the spatial depth layers and $\left(k_{1},k_{2}\right)$ represent discrete angular directions. This tensor serves as a numerical approximation of L(r,σ) and will be used throughout the remainder of this section. Unless otherwise stated, we use $\tilde{L}$ to denote all radiance quantities in the discretized domain.

The core idea of the method is to approximate the RTE using a recursively defined tensor that iteratively computes radiance intensity at discrete steps as a function of depth *r* and direction $\sigma =(\sigma_{1},\sigma_{2})$.

At the initialization stage, we define the first layer:

(13) $${\tilde{L}}_{0}\left(k_{1},k_{2}\right):=L\left(0,\sigma \right),\;\mathrm{where}\;\sigma_{1}=\frac{k_{1}\pi }{n},\;\sigma_{2}=\frac{k_{2}\pi }{2n}.$$

Here, n∈N, $k_{1},k_{2}\in \left\{0,1,\ldots ,n-1\right\}$, and $\sigma \in \left[0,\pi \right]\times \left[0,\frac{\pi }{2}\right]$. This initial matrix represents the radiance in direction σ, as seen in a clear atmosphere, and it thus serves as a fog-free reference image.

Directional sampling in spherical coordinates follows this discretization: $\sigma_{1}=0$ points right, π left, $\sigma_{2}=0$ upward, and π/2 forward. Any arbitrary direction σ can be approximated via the grid $\sigma_{1}=\frac{k_{1}\pi }{n},\sigma_{2}=\frac{k_{2}\pi }{2n}$. In our discretization scheme, not only is the angular domain ω discretized but so is the radial distance in each direction. Specifically, we represent the per-direction distance field as the matrix $d\in R^{n\times n}$, where each entry corresponds to the distance to the object surface in a given sampled direction ω.

We now define tensors indexed by j=1,…,n−1, intended to approximate ${\tilde{L}}_{j}\left(k_{1},k_{2}\right)$, i.e., the radiance after *j* steps. The discretization is bounded as

(14) $$\left|\frac{dj}{n}-r\right|\leq \frac{1}{n},\;\left|\frac{k_{1}\pi }{n}-\sigma_{1}\right|\leq \frac{\pi }{n},\;\left|\frac{k_{2}\pi }{2n}-\sigma_{2}\right|\leq \frac{\pi }{2n}.$$

During the iteration process, we assume that L(r,σ) is sufficiently smooth so that it can be locally approximated by a first-order Taylor expansion:

(15) $$\tilde{L}\left(r,\sigma \right)∼\tilde{L}\left(r-\frac{d}{n},\sigma \right)+\frac{d}{n}\frac{\partial \tilde{L}}{\partial r}\left(r-\frac{d}{n},\sigma \right).$$

Applying the transfer equation from (10), we obtain

(16) $$\begin{array}{cc} \tilde{L}\left(r,\sigma \right) & ∼\tilde{L}\left(r-\frac{d}{n},\sigma \right)+\frac{d}{n}\frac{\partial \tilde{L}}{\partial r}\left(r-\frac{d}{n},\sigma \right)\\ \; & =\tilde{L}\left(r-\frac{d}{n},\sigma \right)\left(1-\frac{d}{n}K\left(r-\frac{d}{n},\sigma \right)\right)\\ \; & +\frac{d}{n}K_{s}\left(r-\frac{d}{n},\sigma \right)4\int_{0}^{\pi }\int_{0}^{\pi /2}\tilde{L}\left(r-\frac{d}{n},\omega \right)\phi \left(\omega ,\sigma \right)\sin\left(\omega_{2}\right)d\omega_{2}d\omega_{1}. \end{array}$$

The final integral term, which represents the accumulated scattered radiance from all directions, is numerically approximated using the following discrete summation:

(17) $$\frac{d}{n}K_{s}\left(\frac{(j-1)d}{n},\sigma \right)\cdot 4\sum_{l_{1}=0}^{n-1}\sum_{l_{2}=0}^{n-1}\frac{\pi }{n}\frac{\pi }{2n}{\tilde{L}}_{j-1}\left(l_{1},l_{2}\right)\;A\left(l_{1},l_{2},k_{1},k_{2}\right),$$

where the tensor $A(l_{1},l_{2},k_{1},k_{2})$ is defined as

(18) $$A\left(l_{1},l_{2},k_{1},k_{2}\right)=\frac{1}{4\pi }\frac{\left(1-g^{2}\right)\sin\left(\frac{\pi l_{2}}{2n}\right)}{B(l_{1},l_{2},k_{1},k_{2})},$$

and the denominator $B(l_{1},l_{2},k_{1},k_{2})$ encodes the components of the Henyey–Greenstein phase function, capturing the anisotropic nature of scattering:

(19) $$\begin{array}{c} B\left(l_{1},l_{2},k_{1},k_{2}\right)\\ ={\left(1-2g\left[\sin\left(\frac{\pi l_{2}}{2n}\right)\sin\left(\frac{\pi k_{2}}{2n}\right)\cos\left(\frac{\pi (l_{1}-k_{1})}{n}\right)+\cos\left(\frac{\pi l_{2}}{2n}\right)\cos\left(\frac{\pi k_{2}}{2n}\right)\right]+g^{2}\right)}^{3/2}. \end{array}$$

This iterative scheme enables the numerical treatment of the general RTE even in the presence of inhomogeneous media and direction-dependent scattering. Thanks to its flexibility, the method is applicable to a wide range of atmospheric radiative transfer problems and provides a more accurate description of light propagation under complex environmental conditions.

By rearranging (17), we obtain the final form of the integral term that will be used within the numerical algorithm:

(20) $$\frac{\pi d}{2n^{3}}K_{s}\left(\frac{(j-1)d}{n},\sigma \right)\left(1-g^{2}\right)\sum_{l_{1}=0}^{n-1}\sum_{l_{2}=0}^{n-1}{\tilde{L}}_{j-1}\left(l_{1},l_{2}\right)\tilde{A}\left(l_{1},l_{2},k_{1},k_{2}\right),$$

where

(21) $$\tilde{A}\left(l_{1},l_{2},k_{1},k_{2}\right)=\frac{\sin\left(\frac{\pi l_{2}}{2n}\right)}{B(l_{1},l_{2},k_{1},k_{2})}.$$

The tensors $A,\tilde{A},B\in R^{n\times n\times n\times n}$ encode spatial interactions between pixel coordinates during scattering computation. The radiance tensor $\tilde{L}$ stores the current estimate of the foggy image intensity values. For grayscale images, $\tilde{L}\in R^{n\times n}$; for color images, the method operates channel-wise, treating $\tilde{L}\in R^{n\times n\times 3}$ as a stack of three independent planes corresponding to the RGB channels. The fog-affected image $\tilde{L}$ is then computed recursively via the following update rule:

(22) $${\tilde{L}}_{j+1}\left(k\right)={\tilde{L}}_{j}\left(k\right)\left(1-\frac{d}{n}K\left(\frac{jd}{n},k\right)\right)+\frac{\pi d}{2n^{3}}K_{s}\left(\frac{jd}{n},k\right)\left(1-g^{2}\right)\sum_{l_{1},l_{2}}{\tilde{L}}_{j}\left(l\right)\tilde{A}\left(l,k\right).$$

Here, $d\in R^{n\times n}$ represents the object distance in the direction characterized by the discrete angle $\sigma =(k_{1}\pi /n,k_{2}\pi /\left(2n\right))$. Since ${\tilde{L}}_{j}$ includes values from directions other than σ, the distance $d_{\sigma }=d_{k}$ may vary during the iteration. As a reminder, the radiance vanishes beyond the visible range: L(r,σ)=0, for $r>d_{\sigma }$.

The above computations can be summarized in the following algorithm (the detailed pseudocode is provided in Appendix A):

1. Choose a resolution parameter n∈N which determines the angular and spatial discretization of the image.
2. Define the distance matrix $d=d_{k}=d\left(k_{1},k_{2}\right)$, which serves as a depth map. Each entry $d_{k}$ represents the distance between the observer and the object in the direction corresponding to $\sigma ∼(k_{1}\pi /n,k_{2}\pi /2n)$.
3. Specify the initial matrix ${\tilde{L}}_{0}\left(k\right)$ of size n×n, which encodes the original radiance intensities in a clear atmosphere. This represents the fog-free reference image.
4. Compute the matrices ${\tilde{L}}_{j}\left(k\right)$ recursively using the following update rule:
  (23) $$\begin{array}{cc} {\tilde{L}}_{j+1}\left(k_{1},k_{2}\right) & ={\tilde{L}}_{j}\left(k_{1},k_{2}\right)\left(1-\frac{d(k_{1},k_{2})}{n}K\right)\\ \; & +\frac{\left(1-g^{2}\right)\pi d\left(k_{1},k_{2}\right)}{2n^{3}}K_{s}\sum_{l_{1}=0}^{n-1}\sum_{l_{2}=0}^{n-1}{\tilde{L}}_{j}\left(l_{1},l_{2}\right)\tilde{A}\left(l_{1},l_{2},k_{1},k_{2}\right). \end{array}$$
5. After the final iteration, the matrix ${\tilde{L}}_{n-1}\left(k\right)$ approximates the fog-attenuated image, i.e., the radiance observed in the direction σ at distance $d(k_{1},k_{2})$.

This algorithm converges to the theoretical solution of the model as n→∞. The functions *K* and $K_{s}$ are assumed to be differentiable and may depend on both the object distance and the wavelength. While there is no direct one-to-one correspondence between physical wavelengths and RGB color channels, both are related to color representation. In our image-based implementation, this dependency is modeled via the RGB channels, using matrix-valued functions as commonly proposed in the literature:

(24) $$K,K_{s}:\left(d,L\right)\to R^{n\times n}.$$

In this study, we validate our method by employing linear element-wise functions:

(25) $$K\left(d,L\right)=A\cdot d+B\cdot L+C,\;K_{s}\left(d,L\right)=X\cdot d+Y\cdot L+Z,$$

where $d,L\in R^{n\times n}$ denote the per-pixel distance and radiance values, $A,B,C,X,Y,Z\in R^{n\times n}$ are constant coefficient matrices, and · denotes the element-wise product.

The overall computational complexity of the original Algorithm A1 is $O(n^{5})$ as each of the $n^{2}$ pixels undergoes a summation over $n^{2}$ directional contributions and *n* recursive steps in the iterative construction and is evaluated across three color channels.

This high computational cost makes the algorithm impractical for real-time or high-resolution applications. Therefore, in the next section, we present algorithmic optimizations to improve performance.

The fourth step of the original algorithm can be reformulated to significantly reduce computational complexity.

Let ${\tilde{L}}_{1},{\tilde{L}}_{2}\in N^{n\times n}$ be defined as

(26) $${\tilde{L}}_{2}:=\left[\begin{array}{cccc} 1 & 2 & \ldots & n\\ \vdots & \vdots & \ddots & \vdots \\ 1 & 2 & \ldots & n \end{array}\right],{\tilde{L}}_{1}:={\tilde{L}}_{2}^{T}.$$

Using these base matrices, we construct the tensors $L_{1},L_{2}\in N^{n\times n\times n\times n}$ by repeating ${\tilde{L}}_{1}$ and ${\tilde{L}}_{2}$ along the third axis *n* times and then replicating the resulting 3D tensors along the fourth axis another *n* times. We also define $K_{1}:=L_{2}^{T}$ and $K_{2}:=L_{1}^{T}$.

The update rule can now be compactly expressed as

(27) $${\tilde{L}}_{j+1}={\tilde{L}}_{j}\cdot \left(1-\frac{K}{n}\cdot d\right)+\frac{\left(1-g^{2}\right)\pi K_{s}}{2n^{3}}\cdot d\cdot \left(\tilde{A}\left(L_{1},L_{2},K_{1},K_{2}\right):{\tilde{L}}_{j}\right),$$

where · denotes element-wise multiplication and : is the tensor double contraction over the third and fourth dimensions:

(28) $$A:B=\sum_{l_{1},l_{2}}A_{l_{1},l_{2},:,:}\;B_{l_{1},l_{2}}.$$

This transformation reduces the iteration complexity from the original $3n^{5}$ steps down to only 3n, offering a major performance boost.

Naturally, this comes at the cost of significantly increased memory usage and moderately higher per-step computation. Nonetheless, further acceleration is achieved by precomputing the tensor $\tilde{A}\left(L_{1},L_{2},K_{1},K_{2}\right)$, eliminating the need to recompute it in each iteration. The trade-off here is that the scattering asymmetry parameter *g* must be fixed in advance.

The complete update procedure is summarized in Algorithm 1, which describes the optimized per-channel evaluation using the precomputed scattering tensor and depth-adaptive extinction. This implementation is used throughout our experiments to simulate fog in a tractable and physically consistent manner.
**Algorithm 1** Optimized Algorithm**Require:** $L,d,n,g,K,K_{s},L_{1},L_{2},K_{1},K_{2}$**Ensure:** $\tilde{L}$$\;\;\;S\leftarrow \tilde{A}\left(L_{1},L_{2},K_{1},K_{2}\right)$$\;\;\;\tilde{L}\leftarrow L$**for** j=0 to n−1 **do**    $L\leftarrow 0^{n\times n\times 3}$    **for** c=0 to 2 **do**        $L\left[:,:,c\right]\leftarrow \tilde{L}\left[:,:,c\right]\cdot \left(1-\frac{K(d,L[:,:,c])}{n}\cdot d\right)+\frac{\left(1-g^{2}\right)\pi K_{s}\left(d,L\left[:,:,c\right]\right)}{2n^{3}}\cdot d\cdot S:\tilde{L}\left[:,:,c\right]$    **end for**    $\tilde{L}\leftarrow L$**end for**

## 4. Dataset

### 4.1. Data Collection and Annotation

To build a diverse and representative dataset for fog synthesis experiments, we collected a total of 25,527 images over a one-month period in April 2022. The images were sourced from 608 publicly available web cameras across 18 countries, predominantly in Europe, with additional contributions from Canada and Russia. The collection captured a wide range of atmospheric conditions and landscapes.

Weather conditions were manually annotated into three primary categories: sunny (1192 images), cloudy (526 images), and foggy (323 images). Night-time and heavily backlit images were excluded to ensure consistent illumination. Also, low-quality images exhibiting artifacts, poor focus, or irrelevant content were removed during a curation phase. After cleaning, the annotated dataset comprised 2041 images. To account for lens distortions, particularly those from fisheye lenses, only central regions of images were retained.

For foggy images, a subjective fog intensity score between 0 and 1 was assigned, representing the perceived density of the fog. This additional annotation served as the foundation for subsequent model training and evaluation.

Additionally, 123 matched foggy-cloudy image pairs were identified across 13 countries, ensuring variability in atmospheric conditions and geographical locations.

### 4.2. Preparation for Model Training

To facilitate domain adaptation for generative models, the fog intensity-annotated images were used for fine-tuning the CycleGAN model. The subjective fog density scores were introduced as an additional conditioning channel during training (see Section 5).

Furthermore, to support the selection of high-quality foggy-cloudy image pairs, an AlexNet-based [24] classifier was trained on the annotated dataset. The classifier achieved 97.1% training accuracy and 85.2% test accuracy, with a weighted test accuracy of 87.7%. By using this model, 123 high-confidence fog-cloudy pairs were extracted, ensuring temporal and spatial consistency for model evaluation.

## 5. Methodology

**Figure 1:**
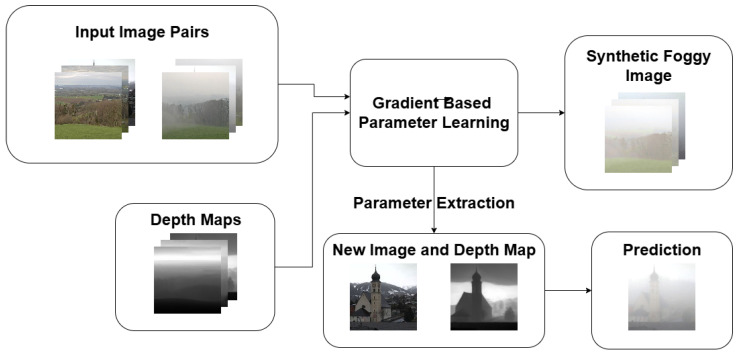
Overview of the training and application workflow of the proposed RTE-based fog synthesis. In the training phase (top), the algorithm received fog–cloudy image pairs with their corresponding depth maps. Starting from randomly initialized matrices, gradient-based optimization was used to learn the extinction and scattering parameters A,B,C and X,Y,Z. In the inference phase (bottom), these learned parameter matrices were reused to synthesize realistic fog on unseen input images based only on their estimated depth maps.

The foggy–cloudy image pairs presented in the previous section were used to calibrate the parameters of the functions K(d,L) and $K_{s}\left(d,L\right)$. In our model, as previously mentioned, extinction *K* and scattering $K_{s}$ coefficients were assumed to be simple linear functions of distance and radiance. This assumption provided a tractable yet flexible parameterization for modeling fog density and light scattering. To this end, we applied a gradient-based optimization approach. The overall training and inference pipeline of our RTE-based fog synthesis framework is illustrated in Figure 1. Our objective was to find the optimal parameter matrices A,B,C,X,Y,Z that minimize the distance *D* between the synthetically generated foggy image $\tilde{L}$ and its real-world counterpart $L_{f}$, thus determining

(29) $$\min_{A,B,C,X,Y,Z}D\left(\tilde{L},L_{f}\right)$$

Let $X,\;Y\in R^{n\times n\times 3}$ denote color images with the respective pixel intensities $X_{i,j,c},Y_{i,j,c}$, where i,j=1,…,n and c=1,2,3 correspond to the three RGB channels.

The Pearson correlation coefficient between two images is defined as

(30) $$\rho \left(X,Y\right)=\frac{\sum_{i=1}^{n}\sum_{j=1}^{n}\sum_{c=1}^{3}\left(X_{i,j,c}-\bar{X}\right)\left(Y_{i,j,c}-\bar{Y}\right)}{\sqrt{\sum_{i=1}^{n}\sum_{j=1}^{n}\sum_{c=1}^{3}{\left(X_{i,j,c}-\bar{X}\right)}^{2}}\;\sqrt{\sum_{i=1}^{n}\sum_{j=1}^{n}\sum_{c=1}^{3}{\left(Y_{i,j,c}-\bar{Y}\right)}^{2}}},$$

where the mean intensities are

(31) $$\bar{X}=\frac{1}{3n^{2}}\sum_{i=1}^{n}\sum_{j=1}^{n}\sum_{c=1}^{3}X_{i,j,c},\;\bar{Y}=\frac{1}{3n^{2}}\sum_{i=1}^{n}\sum_{j=1}^{n}\sum_{c=1}^{3}Y_{i,j,c}.$$

The Pearson distance is then defined as

(32) $$D\left(\tilde{L},L_{f}\right)=1-\rho \left(\tilde{L},L_{f}\right).$$

This scalar quantity considers all pixel values across all three color channels, providing a robust measure of similarity between two images.

We chose the Pearson distance because our algorithm generates a “white fog” effect, which MSE-based metrics can interpret as a deviation from the ground truth image. In contrast, the Pearson distance is invariant to linear brightness shifts, and it therefore does not penalize the lightning effect caused by the synthetic fog.

We used the RMSProp variant of the gradient descent algorithm to optimize the model parameters. Due to the iterative structure of our forward model, the gradient computation also had to follow a recursive approach.

Since ${\tilde{L}}_{n}$ depended on all previous iterations, the total derivative was computed using the chain rule. For a generic parameter U∈A,B,C,X,Y,Z, the following equation was used:

(33) $$\frac{\partial D({\tilde{L}}_{n},L_{f})}{\partial U}=\frac{\partial D({\tilde{L}}_{n},L_{f})}{\partial {\tilde{L}}_{n}}\frac{\partial {\tilde{L}}_{n}}{\partial U}.$$

Thus, the critical component of the optimization was to express ${\tilde{L}}_{n}$ as a function of the learnable parameters. The derivative propagated recursively as follows:

(34) $$\begin{array}{cc} \frac{\partial {\tilde{L}}_{n}}{\partial U} & =\frac{{\tilde{L}}_{n-1}\cdot \left(1-\frac{K(\cdot )}{n}\cdot d\right)+\frac{\left(1-g^{2}\right)\pi K_{s}\left(\cdot \right)}{2n^{3}}\cdot d\cdot \left(\tilde{A}\left(\cdot \right):{\tilde{L}}_{n-1}^{\prime }\right)}{\partial U}\\ \; & =\frac{\partial {\tilde{L}}_{n-1}}{\partial U}\cdot \left(1-\frac{K(\cdot )}{n}\cdot d\right)+{\tilde{L}}_{n-1}\cdot \frac{\partial \left(1-\frac{K(\cdot )}{n}\cdot d\right)}{\partial U}\\ \; & +\frac{\partial \frac{\left(1-g^{2}\right)\pi K_{s}\left(\cdot \right)}{2n^{3}}\cdot d}{\partial U}\cdot \left(\tilde{A}\left(\cdot \right):{\tilde{L}}_{n-1}^{\prime }\right)+\frac{\left(1-g^{2}\right)\pi K_{s}\left(\cdot \right)}{2n^{3}}\cdot d\cdot \tilde{A}\left(\cdot \right):{\left(\frac{\partial {\tilde{L}}_{n-1}}{\partial U}\right)}^{\prime }. \end{array}$$

This process had to be repeated for *n* iterations. Notably, computing $\frac{\partial {\tilde{L}}_{i}}{\partial U}$ at each step required access to all previous derivatives $\frac{\partial {\tilde{L}}_{j}}{\partial U}$ for j<i, but these could be computed in a forward pass and stored efficiently.

The base derivatives for the first iteration were given by

(35) $$\begin{array}{cc} \frac{\partial {\tilde{L}}_{1}}{\partial A} & =-L^{2}\cdot \frac{d}{n},\\ \frac{\partial {\tilde{L}}_{1}}{\partial B} & =-L\cdot \frac{d^{2}}{n},\\ \frac{\partial {\tilde{L}}_{1}}{\partial C} & =-L\cdot \frac{d}{n},\\ \frac{\partial {\tilde{L}}_{1}}{\partial X} & =\frac{(1-g^{2})\pi d}{2n^{3}}\cdot L\cdot A\left(\cdot \right):L,\\ \frac{\partial {\tilde{L}}_{1}}{\partial Y} & =\frac{\left(1-g^{2}\right)\pi d^{2}}{2n^{3}}\cdot A\left(\cdot \right):L,\\ \frac{\partial {\tilde{L}}_{1}}{\partial Z} & =\frac{(1-g^{2})\pi d}{2n^{3}}\cdot A\left(\cdot \right):L. \end{array}$$

Accurate depth estimation was a critical component in our algorithm, as fog density strongly depended on object distance. Obtaining high-quality depth maps proved challenging; to address this, we employed Marigold [25], a state-of-the-art monocular depth estimation model accessible via Hugging Face. While inaccuracies in depth prediction could potentially propagate into artifacts within the synthesized fog distribution, the Marigold model provided sufficiently stable and reliable depth estimates for outdoor scenes, ensuring consistent fog rendering throughout our experiments.

We then fine-tuned the model parameters over 50 training iterations using the previously defined distance metric *D*. The partial derivatives of *D* with respect to ${\tilde{L}}_{n}$ were computed using TensorFlow’s automatic differentiation engine. To reduce memory overhead during training, we manually implemented RMSProp with custom gradient computations based on our Optimized Algorithm with Gradient (see Appendix B). The learning rate was set to α=0.005.

An interesting observation is that by choosing *C* and *Z* as constant scaling parameters and setting all other parameters to zero, our model reduced to the Koschmieder model defined in (11). This enabled a direct comparison between our physically inspired, trainable approach and a widely used analytic model based on homogeneous fog assumptions.

To benchmark our method further against non-physics-based approaches, we also evaluated a generative AI-based fog synthesis model sourced from the GitHub repository *Foggy-CycleGAN* by Zaher Ghais (available at: https://github.com/ghaiszaher/Foggy-CycleGAN; accessed on 7 September 2024). The experiments were conducted using the "2020-06 (legacy)" version of the software, which was the version publicly available at the time. This model was fine-tuned using our custom image dataset. We describe this generative baseline in more detail in the following section.

### CycleGAN

In our experiments, we employed the publicly available Foggy-CycleGAN model [26], originally developed for fog synthesis on clear images from vehicular datasets (Cityscapes [27], SFSU Foggy-Driving [28], RESIDE [29]). Given that these datasets consist of images captured by onboard vehicle cameras, directly applying the pretrained model to our webcam-based outdoor scenes would have introduced a significant domain shift, potentially leading to unfair degradation in performance.

To address this mismatch, the CycleGAN model was fine-tuned using the fog intensity-annotated dataset (see Section 4.2), where each foggy image was assigned a subjective fog density score within the [0,1] interval. The fog density value was introduced as a fourth input channel to condition the generator during training. Importantly, this adaptation was performed solely for experimental consistency and did not involve any architectural modifications to the original CycleGAN framework.

CycleGAN [30] is a well-established generative framework for unpaired image-to-image translation. It leverages adversarial loss and cycle-consistency loss to learn domain mappings. In our application, generators followed a U-Net structure with skip connections, and discriminators adhered to the PatchGAN principle, outputting local authenticity scores.

However, when used for fog synthesis, the model exhibited critical limitations. It generated fog as a style transformation, lacking depth-dependent attenuation and ignoring the directional scattering typical of real fog. As a consequence, fog was often overlaid uniformly across the scene without reflecting variations in object distances and reproducing the forward-scattering characteristics observed in natural conditions. Furthermore, although the intensity conditioning improved some control, the generative process remained stochastic, leading to structural distortions and unpredictable variations in fog appearance.

These deficiencies were confirmed by our experimental evaluation (see Section 6), where the fine-tuned CycleGAN achieved higher Fréchet Inception Distance (FID) and lower Pearson correlation values compared to our proposed RTE-based method, confirming its limited capability in generating physically realistic and structure-preserving fog.

In summary, although CycleGAN provides a flexible and efficient framework for general visual translation, it is inherently inadequate for tasks requiring physically accurate atmospheric effects. This motivates the need for physics-driven alternatives such as the discretized Radiative Transfer Equation (RTE) approach introduced in this study.

## 6. Experimental Results

**Figure 2:**
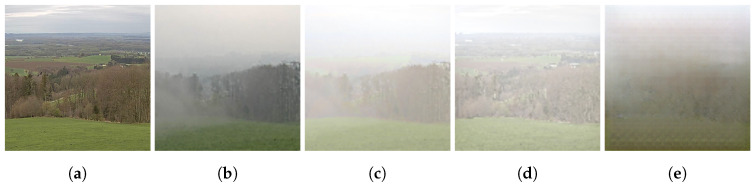
A visual comparison of synthetic fog generated by different models. The proposed RTE-based method (**c**) produces fog effects that are visually more consistent with the real-world reference (**b**), capturing both depth-aware attenuation and directional scattering: (**a**) cloudy image (input); (**b**) real fog (ground truth); (**c**) proposed RTE-based model; (**d**) Koschmieder’s model; (**e**) CycleGAN output.

To provide a visual comparison of the different fog generation methods, Figure 2 shows sample outputs from each approach. The input image (a) is compared to the real foggy counterpart (b), along with the synthesized outputs from the proposed RTE-based model (c), the classical Koschmieder model (d), and the CycleGAN approach (e). Notably, the proposed method produces fog that visually resembles the real reference image more closely, especially in terms of depth-dependent attenuation and spatial heterogeneity.

To evaluate the realism and quality of the fog produced by the three models, we used the Fréchet Inception Distance (FID) metric [31]. In addition, we report the Pearson correlations between the mean feature vectors used in the FID formula.

There are two motivations for this. First, to visualize the similarity between the images in vector form, we reduced the foggy, cloudy, and sunny images’ mean vectors to 2D via Principal Component Analysis (PCA). Second, since the FID metric places significant weight on the covariance matrix, this could distort results given the small number of foggy–cloudy image pairs in our dataset.

Table 1 and Table 2 present a comparative analysis of the proposed method and baseline approaches using standard image quality metrics such as the FID and the Pearson correlation. To facilitate comparison, the FID values were normalized to 1. The results demonstrate that our approach consistently yielded improved visual quality across all evaluated scenes.

**Figure 3:**
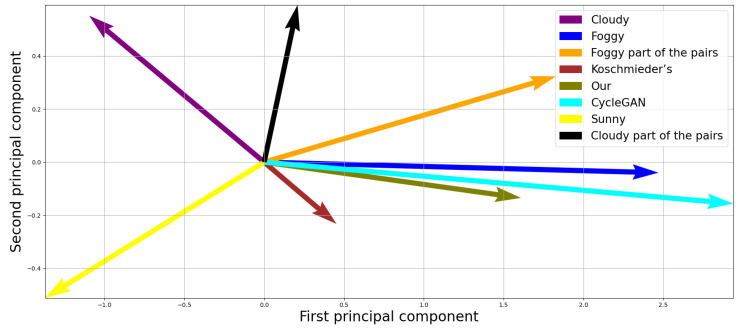
A 2D visualization of the mean feature vectors.

Our proposed model achieved the highest similarity to real foggy images across both metrics (the Pearson correlation and the FID). Koschmieder’s classical model ranked second, offering moderate fidelity, while the CycleGAN approach exhibited the weakest results, with the highest FID and lowest correlation values. Interestingly, despite being trained on the same dataset, the CycleGan tended to generate a more generalized and stylized form of fog. This tendency becomes apparent in the 2D vector visualization, as shown in Figure 3, where the cyan vector representing the CycleGan output may initially appear to be the most visually appealing. However, the vector corresponding to our model (olive) is closely aligned in terms of direction, indicating that it captures similarly realistic fog characteristics.

A key distinction lies in the fact that our method explicitly utilizes paired foggy–cloudy images during training, whereas the CycleGAN does not incorporate such pairing constraints. As a result, the fog produced by our model is visually consistent and semantically aligned with the structure of real-world transformations. In contrast, the Koschmieder model generates fog that deviates significantly in representation space, suggesting a fundamentally different—more theoretical and homogeneous—fog structure.

In summary, our physically informed model can generate realistic and spatially varying fog that closely mimics certain natural fog types. While the Koschmieder model does achieve domain translation in a technical sense, its results fall short in terms of both realism and structural fidelity. The CycleGAN, although capable of generating visually plausible fog, underperforms in terms of FID and correlation, likely due to its inherently synthetic nature and the absence of paired supervision.

### Sensitivity to Depth Estimation Noise

To evaluate the robustness of our fog synthesis model to depth estimation inaccuracies, we performed a controlled noise analysis. A subset of 40 fog–cloudy image pairs was selected, and Gaussian noise was injected into the distance matrix *d*. Specifically, we tested zero-mean normal distributions with the standard deviations σ=0.05 and σ=0.10, representing 5% and 10% perturbations relative to normalized depth values.

After recomputing the synthetic foggy images with the noisy distance maps, we measured the degradation in image quality using both the FID and the Pearson correlation. Table 3 summarizes the results.

As the results show, depth noise leads to a gradual decline in both perceptual and structural fidelity. However, even with 10% noise, the model retains a high level of plausibility, suggesting that it is relatively robust to the kinds of moderate errors typically observed in monocular depth prediction.

## 7. Discussion

Our experiments revealed several key insights regarding the practical applicability and performance of the proposed RTE-based fog synthesis framework.

First, the method exhibited notable memory demands, primarily due to the use of large angular and spatial tensors. To quantify this, we evaluated the model at various image resolutions in both training and inference modes. Table 4 summarizes the runtime and VRAM usage during foggy image generation, while Table 5 reports the same during a single training iteration.

In comparison, the Koschmieder model—due to its closed-form nature—requires negligible time and memory. The CycleGAN model is also significantly faster during inference: On an NVIDIA RTX 3090 GPU, fog generation takes only 0.037 s per 256 × 256 image with a VRAM footprint of  1.5 GB. However, this comes at the cost of long training times, often requiring multiple days of GPU computation and at least 8 GB of memory. Moreover, training instability, mode collapse, and overfitting are well-known challenges in GAN-based systems.

In our experiments, we observed additional limitations of GAN training:

- Training instability:The adversarial nature of generator–discriminator optimization often led to oscillations or collapse;
- Artifact generation: At higher fog densities, GAN outputs suffered from color shifts and visual artifacts;
- Poor generalization: The pretrained CycleGAN was biased toward in-vehicle urban scenes and failed to generalize to diverse landscapes.

Although the number of fog–cloudy pairs in our dataset is relatively small (123), we investigated the generalizability of the model beyond the training set. To this end, we split the data into 40 training and 83 test pairs and performed fog synthesis on the test images using parameters averaged over the training subset. Despite the lack of image-specific optimization, the generated foggy images achieved a Pearson correlation of 0.889 and an FID score of 0.641—values comparable to those observed during training and superior to those of the CycleGAN baseline.

These findings indicate that despite being trained on limited paired data, the RTE-based model shows potential for broader applicability. In addition to parameter averaging, future extensions could explore selection-based parameter reuse, that is, applying the physical coefficients K(d,L) and $K_{s}\left(d,L\right)$ from the most similar training image(s). Similarity could be assessed using depth distribution statistics, such as histogram distances or divergence measures. This approach may enable data-efficient, adaptive application of the model to unseen scenes without full retraining.

Regarding parameter sensitivity, we observed that the depth map quality is paramount. The pretrained Marigold model provided sufficiently accurate monocular depth estimates, which proved critical for spatially coherent fog effects. Among the learnable parameters, the extinction coefficient had the strongest influence on visual realism, primarily modulating the intensity of multi-scattering.

After manual tuning, we found that 50 iterations consistently minimized error across all images when using RMSProp. This balance between optimization depth and computational cost was essential for scalability. At higher iteration counts, we occasionally encountered issues with exploding gradients, which further motivated the choice of 50 as a practical upper bound.

A major strength of our method is its physical generality: it can readily be adapted to simulate other atmospheric media, such as dust, smog, or smoke, without fundamental changes to the model structure. Although sensitive to depth map noise, as discussed in Section 6, the method remains robust under realistic perturbations and is well-suited for augmenting clear-weather datasets with physically plausible fog effects. This opens the door to training perception systems under degraded visibility conditions in safety-critical domains such as autonomous driving.

## 8. Conclusions

This study proposed a novel, physically grounded framework for image-space fog synthesis based on the discretization of the Radiative Transfer Equation (RTE). Unlike traditional models that assume homogeneous scattering or rely solely on Generative Adversarial Networks, our method accurately captures inhomogeneous and anisotropic fog behaviors by integrating scene geometry and light transport physics.

The key strength of our approach lies in its ability to generate visually and structurally consistent fog effects by optimizing learnable extinction and scattering parameters, calibrated on real foggy–cloudy image pairs. Quantitative results confirmed superior performance over both Koschmieder’s analytic model and CycleGAN-based baselines, validating our model’s capability to bridge physical accuracy with perceptual realism.

However, the method presents challenges in terms of memory usage and runtime complexity, particularly during training. Although the current implementation already relies on TensorFlow tensor representations, the memory footprint becomes significant as the angular resolution increases. For instance, memory usage exceeding 24 GB highlights the need for more compact representations or structured sparsity in future designs.

In addition to direct improvements of the current framework, we also propose a new research direction: leveraging our physically grounded RTE-based model to train or constrain a Generative Adversarial Network. In particular, we aim to develop a hybrid pipeline where a GAN learns to approximate the output of the RTE simulator, conditioned on depth and image data. This would combine the interpretability and physical consistency of our model with the generative efficiency of neural architectures. Furthermore, we plan to integrate this with monocular depth estimation models such as Marigold, creating an end-to-end differentiable system for photorealistic fog generation.

Crucially, the model’s modular design and reliance on real image data make it well-suited for data augmentation in machine learning pipelines, particularly in safety-critical domains such as autonomous driving. By converting clear-weather footage into foggy scenarios with physical plausibility, our framework enables realistic training environments under degraded visibility conditions.

In summary, this work demonstrates the feasibility and utility of using discretized RTE for controllable, structure-aware fog synthesis. We hope it paves the way for the future integration of physics-based modeling and learning-based adaptation in environmental simulation tasks.

## Figures and Tables

**Table 1:** Normalized FID values.

	Fog	Fog Pair	Our	Koschmieder’s	CycleGAN
Fog	-	0.399	0.512	0.57	0.785
Fog pair	0.399	-	0.327	0.448	0.867
Cloudy	0.535	0.58	0.638	0.577	0.979
Sunny	0.553	0.618	0.654	0.58	1.0

**Table 2:** Pearson correlations between image groups and model outputs.

	Fog	Fog Pair	Our	Koschmieder’s	CycleGAN
Fog	-	0.964	0.926	0.887	0.765
Fog pair	0.964	-	0.953	0.929	0.759
Cloudy	0.864	0.893	0.858	0.909	0.589
Sunny	0.844	0.872	0.848	0.909	0.567

**Table 3:** The impact of Gaussian noise in depth estimation on fog synthesis quality. All metrics are averaged over 40 images.

Noise std σ	FID	Pearson	ΔCorrelation (%)
0 (baseline)	1.00	0.937	–
0.05	1.093	0.928	−1.0%
0.10	1.162	0.910	−2.9%

**Table 4:** Average inference time and VRAM usage for RTE-based fog generation at different resolutions (in seconds and MB).

Resolution (px)	Avg Time (s)	Avg VRAM (MB)
25 × 25	0.115	330
50 × 50	0.266	444
75 × 75	0.870	960
100 × 100	2.515	2366
125 × 125	6.150	5392
150 × 150	13.302	10,960
175 × 175	25.550	18,356

**Table 5:** Average training time and VRAM usage per iteration (RMSProp) for RTE model.

Resolution (px)	Avg Iteration Time (s)	Avg VRAM (MB)
25 × 25	0.661	1133
50 × 50	2.001	1247
75 × 75	9.469	1754
100 × 100	30.073	3197
125 × 125	77.633	6323
150 × 150	169.786	11,874
175 × 175	330.760	21,173

## Data Availability

The data used in this study were obtained from publicly accessible online webcam streams indexed on the http://www.insecam.org/en/ (accessed between 1 and 20 April 2022) platform. Due to the distributed and dynamic nature of the sources, direct URLs are not included in the article. The images are not publicly shared, but a list of source URLs and example frames can be provided by the corresponding author upon reasonable request. The data were collected solely for non-commercial academic research purposes.

## Appendix A. Pseudocode of the Original Algorithm

For completeness, the pseudocode of the original, non-optimized algorithm is given below. This algorithm corresponds to the sequential execution of the steps described in Section 3.2.

**Algorithm A1** Original Algorithm
**Require:** $L,d,n,g,K,K_{s}$ **Ensure:** $\tilde{L}$ $\;\;\;\tilde{L}\leftarrow L$ **for** j=0 to n−1 **do** $L\leftarrow 0^{n\times n\times 3}$ **for** c=0 to 2 **do** **for** $k_{1}=0$ to n−1 **do** **for** $k_{2}=0$ to n−1 **do** S←0 **for** $l_{1}=0$ to n−1 **do** **for** $l_{2}=0$ to n−1 **do** $S\leftarrow S+\tilde{A}\left(k_{1},k_{2},l_{1},l_{2}\right)\tilde{L}\left[l_{1},l_{2},c\right]$ **end for** **end for** $\begin{array}{c} L[k_{1},k_{2},c]\leftarrow \tilde{L}\left[k_{1},k_{2},c\right]\left(1-\frac{d[k_{1},k_{2}]}{n}K\left(d,\tilde{L}\left[:,:,c\right]\right)\left[k_{1},k_{2}\right]\right)\\ \;\;\;\;+\frac{\left(1-g^{2}\right)\pi d\left[k_{1},k_{2}\right]}{2n^{3}}K_{s}\left(d,\tilde{L}\left[:,:,c\right]\right)\left[k_{1},k_{2}\right]S \end{array}$ **end for** **end for** **end for** $\tilde{L}\leftarrow L$ **end for**

## Appendix B. Optimized Algorithm with Gradient

This appendix provides the full pseudocode of the optimized fog synthesis algorithm that incorporates gradient-based improvements. This implementation corresponds to the enhanced version described in Section 5, where parameter updates are performed using RMSProp and custom recursive differentiation. The algorithm includes the efficient handling of tensor operations, the precomputation of the scattering kernel, and gradient propagation with respect to all learnable physical parameters. This detailed formulation is also useful for reproducing the optimization process and understanding the implementation-level considerations of our RTE-based framework.

**Algorithm A2** Optimized Algorithm with Gradient
**Require:** $L,d,n,g,K,K_{s},L_{1},L_{2},K_{1},K_{2}$ **Ensure:** $\tilde{L},\frac{\partial \tilde{L}}{\partial A},\frac{\partial \tilde{L}}{\partial B},\frac{\partial \tilde{L}}{\partial C},\frac{\partial \tilde{L}}{\partial X},\frac{\partial \tilde{L}}{\partial Y},\frac{\partial \tilde{L}}{\partial Z}$ Initialize: $$\begin{array}{c} S\leftarrow \tilde{A}\left(L_{1},L_{2},K_{1},K_{2}\right),\tilde{L}\leftarrow L,\\ {\tilde{L}}_{\partial A_{0}}\leftarrow -L^{2}\cdot \frac{d}{n},\;{\tilde{L}}_{\partial B_{0}}\leftarrow -L\cdot \frac{d^{2}}{n},\;{\tilde{L}}_{\partial C_{0}}\leftarrow -L\cdot \frac{d}{n},\\ {\tilde{L}}_{\partial X_{0}}\leftarrow \frac{(1-g^{2})\pi d}{2n^{3}}\cdot L\cdot S:L,\\ {\tilde{L}}_{\partial Y_{0}}\leftarrow \frac{\left(1-g^{2}\right)\pi d^{2}}{2n^{3}}\cdot S:L,\\ {\tilde{L}}_{\partial Z_{0}}\leftarrow \frac{(1-g^{2})\pi d}{2n^{3}}\cdot S:L. \end{array}$$ **for** j=0 to n−1 **do** Initialize $L\leftarrow 0^{n\times n\times 3}$ **for** c=0 to 2 **do** Compute the updated $\tilde{L}$: $$\begin{array}{ll} L\left[:,:,c\right]\leftarrow \tilde{L}\left[:,:,c\right]\cdot & \left(1-\frac{K(d,L)}{n}\cdot d\right)\\ & +\frac{\left(1-g^{2}\right)\pi K_{s}\left(d,L\right)}{2n^{3}}\cdot d\cdot S:\tilde{L}\left[:,:,c\right]. \end{array}$$ Compute gradients: $$\begin{array}{ll} {\tilde{L}}_{\partial A}\left[:,:,c\right]\leftarrow {\tilde{L}}_{\partial A_{0}}\left[:,:,c\right]\cdot & \left(1-\frac{K(d,L)}{n}\cdot d\right)-\frac{d}{n}\cdot L\left[:,:,c\right]\cdot \left(L\left[:,:,c\right]+A\cdot {\tilde{L}}_{\partial A_{0}}\left[:,:,c\right]\right)\;\\ & +\frac{\left(1-g^{2}\right)\pi X\cdot {\tilde{L}}_{\partial A_{0}}\left[:,:,c\right]}{2n^{3}}\cdot d\cdot S:L\left[:,:,c\right]\\ & +\frac{\left(1-g^{2}\right)\pi K_{s}\left(d,L\right)}{2n^{3}}\cdot d\cdot S:{\tilde{L}}_{\partial A_{0}}\left[:,:,c\right], \end{array}$$ $$\begin{array}{ll} {\tilde{L}}_{\partial X}\left[:,:,c\right] & \leftarrow {\tilde{L}}_{\partial X_{0}}\left[:,:,c\right]\cdot \left(1-\frac{K(d,L)}{n}\cdot d\right)-\frac{d}{n}\cdot L\left[:,:,c\right]\cdot \left(A\cdot {\tilde{L}}_{\partial X_{0}}\left[:,:,c\right]\right)\\ & +\frac{\left(1-g^{2}\right)\pi \left(L\left[:,:,c\right]+X\cdot {\tilde{L}}_{\partial X_{0}}\left[:,:,c\right]\right)}{2n^{3}}\cdot d\cdot S:L\left[:,:,c\right]\\ & +\frac{\left(1-g^{2}\right)\pi K_{s}\left(d,L\right)}{2n^{3}}\cdot d\cdot S:{\tilde{L}}_{\partial X_{0}}\left[:,:,c\right]. \end{array}$$ Apply similar updates for ${\tilde{L}}_{\partial B}$, ${\tilde{L}}_{\partial C}$, ${\tilde{L}}_{\partial Y}$, and ${\tilde{L}}_{\partial Z}$. **end for** Update: $$\begin{array}{c} \;\;\tilde{L}\leftarrow L,\\ {\tilde{L}}_{\partial A_{0}}\leftarrow {\tilde{L}}_{\partial A},\;{\tilde{L}}_{\partial B_{0}}\leftarrow {\tilde{L}}_{\partial B},\;{\tilde{L}}_{\partial C_{0}}\leftarrow {\tilde{L}}_{\partial C},\\ {\tilde{L}}_{\partial X_{0}}\leftarrow {\tilde{L}}_{\partial X},\;{\tilde{L}}_{\partial Y_{0}}\leftarrow {\tilde{L}}_{\partial Y},\;{\tilde{L}}_{\partial Z_{0}}\leftarrow {\tilde{L}}_{\partial Z}. \end{array}$$ **end for**
